# DCE-MRI-based radiomics in predicting angiopoietin-2 expression in hepatocellular carcinoma

**DOI:** 10.1007/s00261-023-04007-8

**Published:** 2023-07-26

**Authors:** Jing Zheng, Pei-Zhuo Du, Cui Yang, Yun-Yun Tao, Li Li, Zu-Mao Li, Lin Yang

**Affiliations:** 1 Medical Imaging Key Laboratory of Sichuan Province, Department of Radiology, Interventional Medical Center, Medical Research Center, Affiliated Hospital of North Sichuan Medical College, Nanchong, 637000 China; 2 Department of Radiology, The Second Clinical Medical College of North Sichuan Medical College, Nanchong, 637000 China; 3 Department of Radiology, Panzhihua Central Hospital, Panzhihua, 617000 China; 4 Department of Pathology, Affiliated Hospital of North Sichuan Medical College, Nanchong, 637000 China

**Keywords:** Radiomics, MRI, Hepatocellular carcinoma, Angiogenesis

## Abstract

**Background:**

Hepatocellular carcinoma (HCC) is the sixth most common cancer, and the third leading cause of cancer death worldwide. Studies have shown that increased angiopoietin-2 (Ang-2) expression relative to Ang-1 expression in tumors is associated with a poor prognosis.The purpose of this study was to investigate the efficacy of predicting Ang-2 expression in HCC by preoperative dynamic contrast‐enhanced magnetic resonance imaging (DCE-MRI)-based radiomics.

**Methods:**

The data of 52 patients with HCC who underwent surgical resection in our hospital were retrospectively analyzed. Ang-2 expression in HCC was analyzed by immunohistochemistry. All patients underwent preoperative upper abdominal DCE-MRI and intravoxel incoherent motion diffusion-weighted imaging scans. Radiomics features were extracted from the early and late arterial and portal phases of axial DCE-MRI. Univariate analysis and least absolute shrinkage and selection operator (LASSO) was performed to select the optimal radiomics features for analysis. A logistic regression analysis was performed to establish a DCE-MRI radiomics model, clinic-radiologic (CR) model and combined model integrating the radiomics score with CR factors. The stability of each model was verified by 10-fold cross-validation. Receiver operating characteristic (ROC) curve analysis, calibration curve analysis and decision curve analysis (DCA) were employed to evaluate these models.

**Results:**

Among the 52 HCC patients, high Ang-2 expression was found in 30, and low Ang-2 expression was found in 22. The areas under the ROC curve (AUCs) for the radiomics model, CR model and combined model for predicting Ang-2 expression were 0.800, 0.874, and 0.933, respectively. The DeLong test showed that there was no significant difference in the AUC between the radiomics model and the CR model (*p* > 0.05) but that the AUC for the combined model was significantly greater than those for the other 2 models (*p *< 0.05). The DCA results showed that the combined model outperformed the other 2 models and had the highest net benefit.

**Conclusion:**

The DCE-MRI-based radiomics model has the potential to predict Ang-2 expression in HCC patients; the combined model integrating the radiomics score with CR factors can further improve the prediction performance.

**Graphical abstract:**

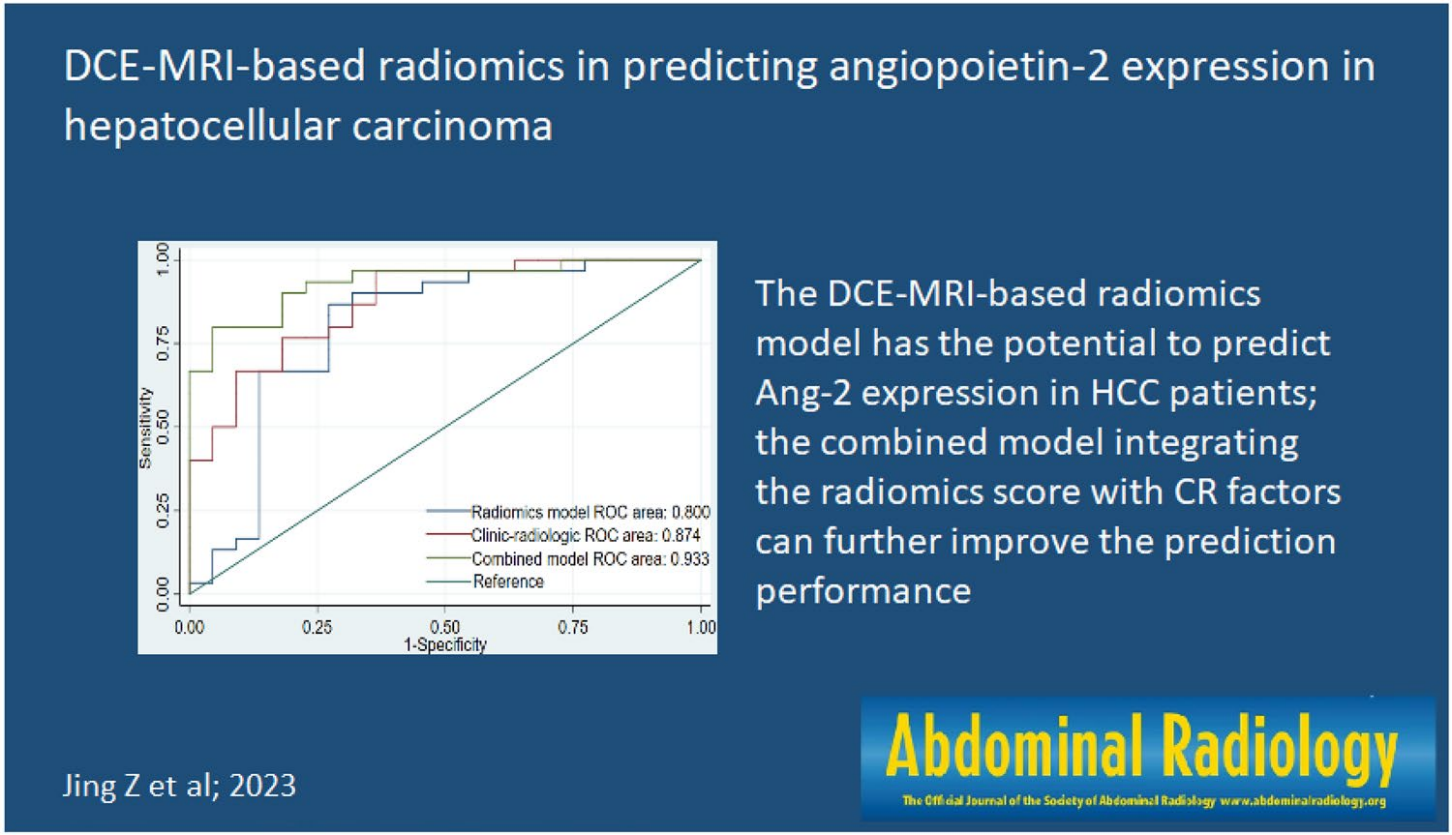

## Introduction

Hepatocellular carcinoma (HCC) is the sixth most common cancer, and the third leading cause of cancer death worldwide [1]. Tumor biological behavior is increasingly recognized as an important factor affecting prognosis. Tumor angiogenesis is the result of the complex interaction among various proangiogenic mediators and effector cells [2]. The rapid deepening of the understanding of the molecular mechanisms of angiogenesis has led to the emergence of antiangiogenic drugs to treat cancer, with thousands of patients benefitting from vascular endothelial growth factor (VEGF) and angiopoietin inhibitors [3, 4]. Angiopoietins are ligands for the endothelial cell receptor Tie-2 and play an important role in tumor angiogenesis. Studies have shown that increased angiopoietin-2 (Ang-2) expression relative to Ang-1 expression in tumors is associated with a poor prognosis [5]. Ang-2 expression in tumor cells decreases after lenvatinib treatment, as anti-VEGF treatment is thought to reduce Ang-2 expression in tumor cells by normalizing tumor blood vessels and reducing hypoxia in the tumor microenvironment [6, 7]. Early changes in Ang-2 levels may help predict clinical efficacy and progression-free survival (PFS) in HCC patients treated with lenvatinib [8]. Therefore, detecting the expression of Ang-2 in HCC tissue could be helpful for predicting prognosis and formulating a targeted therapy strategy.

Imaging features can serve as molecular surrogates for diagnosis and prognosis and the evaluation of possible gene expression-related therapeutic responses in various human cancers [9]. Le Bihan al. [10, 11] proposed intravoxel incoherent motion diffusion-weighted imaging (IVIM-DWI), which can be used to noninvasively observe the microstructure of human tissue by distinguishing the diffusion of water molecules from the perfusion of microvessels through a biexponential model. In recent years, Dutch scholars Lambin et al. [12] proposed the concept of radiomics, which is the automatic high-throughput extraction of many image features based on image analysis. Solid tumors have different spatial and temporal heterogeneities at different levels, limiting the use of biopsy-based molecular assays but offering enormous opportunities for noninvasive imaging radiomics [13].

To date, there has been no report on predicting Ang-2 expression in HCC tissue by a dynamic contrast‐enhanced magnetic resonance imaging (DCE-MRI)-based model. Therefore, this study investigated the efficacy of preoperative DCE-MRI-based radiomics in predicting Ang-2 expression in HCC.

## Materials and methods

### Patients

Fifty-two patients with HCC who underwent surgical resection were enrolled. All patients underwent preoperative upper abdominal DCE-MRI scans and IVIM-DWI scans. The inclusion criteria were as follows: HCC confirmed by pathological examination; no antitumor therapy; liver lesions > 1 cm; and MRI scan within one week before surgery. The exclusion criteria were as follows: MRI contraindications and poor image quality that affects image segmentation. Clinical characteristics that may be associated with Ang-2 expression were collected. Laboratory indicators were classified into categorical variables based on a threshold value.

### Immunohistochemical detection

Ang-2 antibody was obtained from Abcam, UK, and used at a dilution of 1:250. Specimens were obtained from histopathologically confirmed HCC patients who underwent MRI examination and surgical resection in our hospital. S-P immunohistochemical staining was performed to score the immunohistochemical staining results. A diagnostic evaluation of the same section was performed by 2 physicians. When there was a disagreement, a conclusion was made after discussion. The degrees of staining under 6 high-power fields were recorded for each section. Ang-2 protein expression intensity was scored as follows: 0 (no staining), 1 (weak staining, light yellow), 2 (moderate staining, brown), and 3 (strong staining, yellowish brown). Scores of 0 and 1 were considered low expression, and scores of 2 and 3 were considered high expression [14].

### MRI scan

**Fig. 1:**
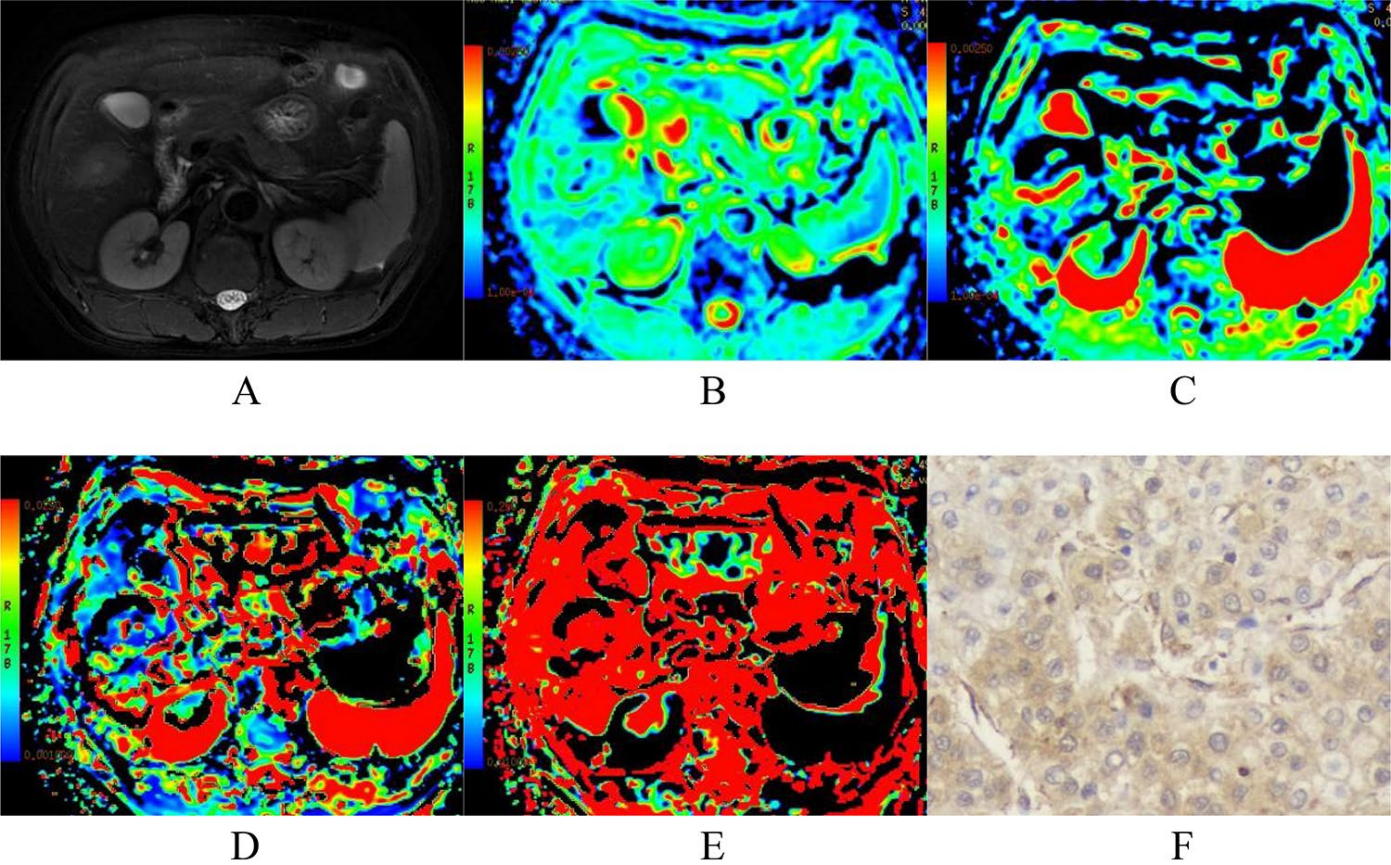
A 63-year-old male with HCC. **a** Image of T2WI, **b** Image of ADC, **c** Image of D, **d** Image of D^*^, **e** Image of f, **f** Immunohistochemistry showed that Ang-2 was highly expressed (× 200).

A Discovery 750 3.0-T superconducting MRI scanner (GE, United States) with a 32-channel phased‐array receiver coil was used. The sequences were as follows: breath-hold transverse-axis fat-suppression T1 weighted imaging (T1WI) scans and breath-triggered transverse-axis fat-suppression T2WI and IVIM-DWI scans. The T1WI sequence parameters were as follows: repetition time (TR)/echo time (TE), 4 ms/2 ms; fractional anisotropy (FA), 12; matrix, 260 × 192; field of view (FOV), 36 cm × 36 cm–40 cm × 40 cm; and slice thickness/interslice gap, 5 mm/0 mm. The T2WI sequence parameters were as follows: TR/TE, 2609 ms/97 ms; FA, 110.0; matrix, 384 × 384; FOV, 36 cm × 36 cm–40 cm × 40 cm; and slice thickness/interslice gap, 5 mm/1 mm. The IVIM-DWI sequence parameters were as follows: 9 *b* values (*b *= 0, 20, 50, 100, 150, 200, 400, 800, and 1000); TR/TE, 3529 ms/60.8 ms; matrix, 128 × 160; FOV, 36 cm × 36 cm–40 cm × 40 cm; and slice thickness/interslice gap 5 mm/1 mm. For multiphase DCE-MRI scanning, a high-pressure syringe was used to inject the contrast agent Gd-DTPA (15–20 ml) into the dorsal vein of the hand; the injection rate was 2–2.5 ml/s. Then, early and late arterial and portal phase images were collected. Using the Function-MADC model of the GE AW 4.4 workstation on the measured image data, the best tumor slice of the IVIM-DWI sequence was selected, the region of interest (ROI) was manually delineated, and the pseudocolor images of IVIM-DWI parameters, including the apparent diffusion coefficient (ADC), slow apparent diffusion coefficient (D), fast apparent diffusion coefficient (D^*^), and fraction of fast apparent diffusion coefficient (f), were generated (Fig. 1), thus obtaining the ADC value, *D* value, D^*^ value, and f value. When delineating the ROIs, areas of hemorrhage, necrosis, cystic degeneration and fat were avoided as much as possible. Each parameter was measured 3 times, and the average was taken. Univariate analysis was used to evaluate clinic-radiologic (CR) factors that could potentially differentiate high and low Ang-2 expression in the cohort. Important variables in the univariate analysis were entered into the multivariate logistic regression analysis, and the potential predictors for high Ang-2 expression were screened out.

### Lesion segmentation and feature extraction

**Fig. 2:**
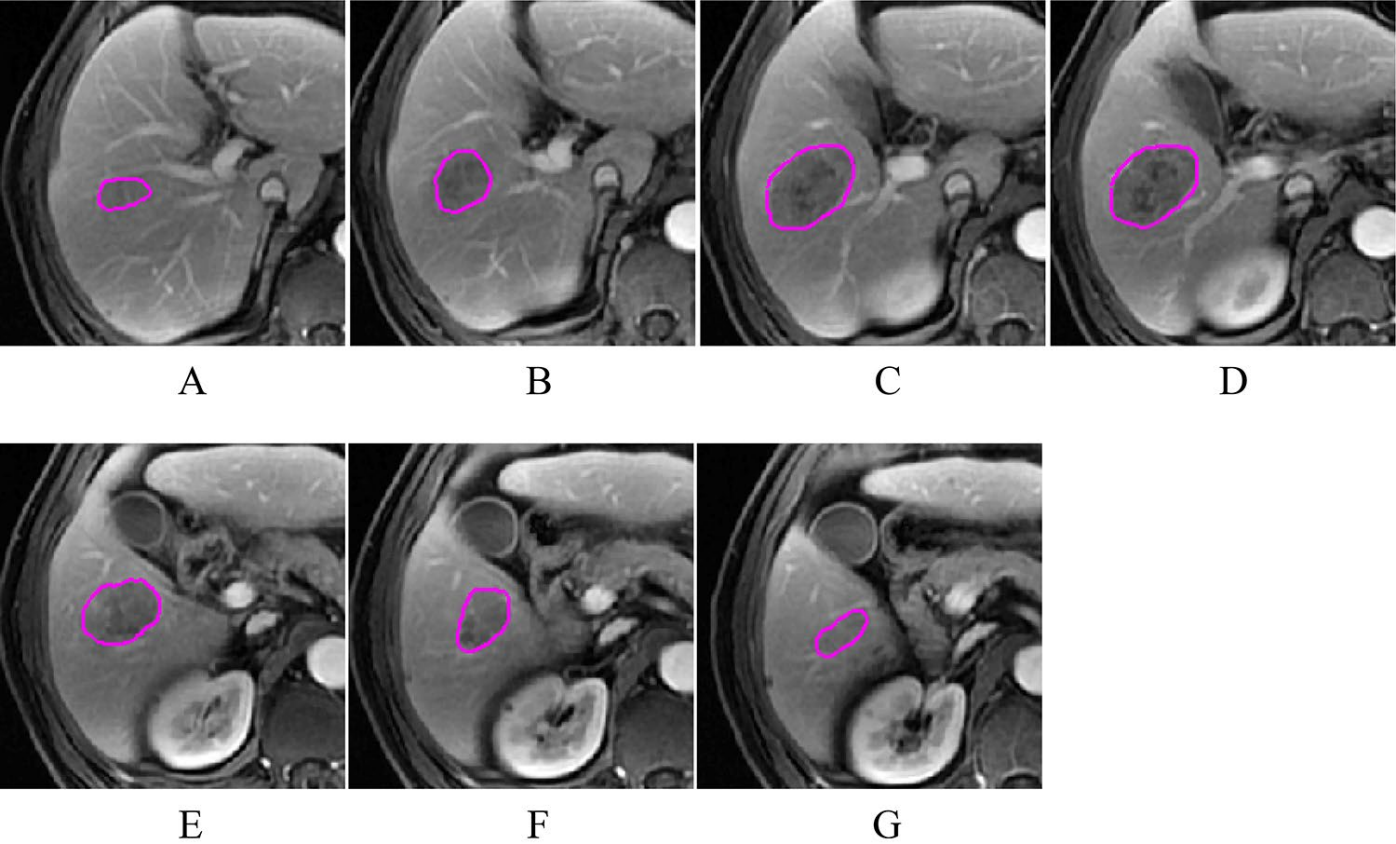
The region of interest (ROI) was delineated layer by layer (**a**–**g**) on the DCE-MRI images to obtain the volume of interest (VOI) for radiomics feature extraction.

Images in digital imaging and communications in medicine (DICOM) format obtained from DCE-MRI scans were exported from the picture archiving and communication system (PACS). Image segmentation was performed manually [15] (Fig. 2) by two radiologists (Observer 1, T.Y.Y. and Observer 2, Z.J., with 4 and 6 years of experience in abdominal MRI imaging interpretation, respectively). Each ROI covered the entire tumor, including all areas of hemorrhage or necrosis in the tumor and avoiding areas of peritumoral edema and obvious large blood vessel invasion [16]. ROIs were delineated layer by layer on the three-phase DCE-MRI images to obtain the volume of interest (VOI) for radiomics feature extraction. This study extracted 4 types of features: shape, intensity histogram, gray level co-occurrence matrix (GLCM), and gray level run length matrix (GLRLM) features.

The consistency analyses in this study included intraobserver and interobserver consistency tests. Observer 1 delineated the ROIs of all images layer by layer in accordance with the delineation method described above after 1 week and extracted features. The extracted features were compared with the results of the first delineation, and the intraclass correlation coefficient (ICC) of the intraobserver consistency was obtained. Observer 2 independently delineated the ROIs in the same way as described above, and the features were compared with those extracted by Observer 1 to obtain the interclass ICC of interobserver consistency. Intragroup ICC/intergroup ICC values ≥ 0.75 indicated good agreement.

### Model establishment and evaluation

After the consistency test, for the radiomics features with ICC values ≥ 0.75, the missing values were filled, and to ensure the reproducibility of the results, z score normalization was performed on all data as a preprocessing step. For feature dimensionality reduction, univariate analysis was used to select the features with statistically significant differences in Ang-2 expression. Least absolute shrinkage and selection operator (LASSO) regression analysis was used to reduce the dimensionality of the features. Binary logistic regression analysis was performed to establish a DCE-MRI radiomics model, CR model, and combined model using the selected radiomics features and CR parameters. The stability of the model was verified by 10-fold cross-validation, and calibration curves were used to evaluate the 3 models. Receiver operating characteristic (ROC) curves were used to evaluate the predictive performance of the 3 models. The evaluation indicators included the area under the curve (AUC), accuracy, sensitivity, and specificity, and calibration curve analysis and decision curve analysis (DCA) were used to evaluate the models.

### Statistical analysis

The statistical software R (Version 4.0.3) was used for statistical analysis in this study. Continuous variables were analyzed using the independent samples *t* test or Kruskal‒Wallis nonparametric rank-sum test; categorical variables were analyzed using the chi-square test or Fisher's exact test.

## 3. Results

**Fig. 3:**
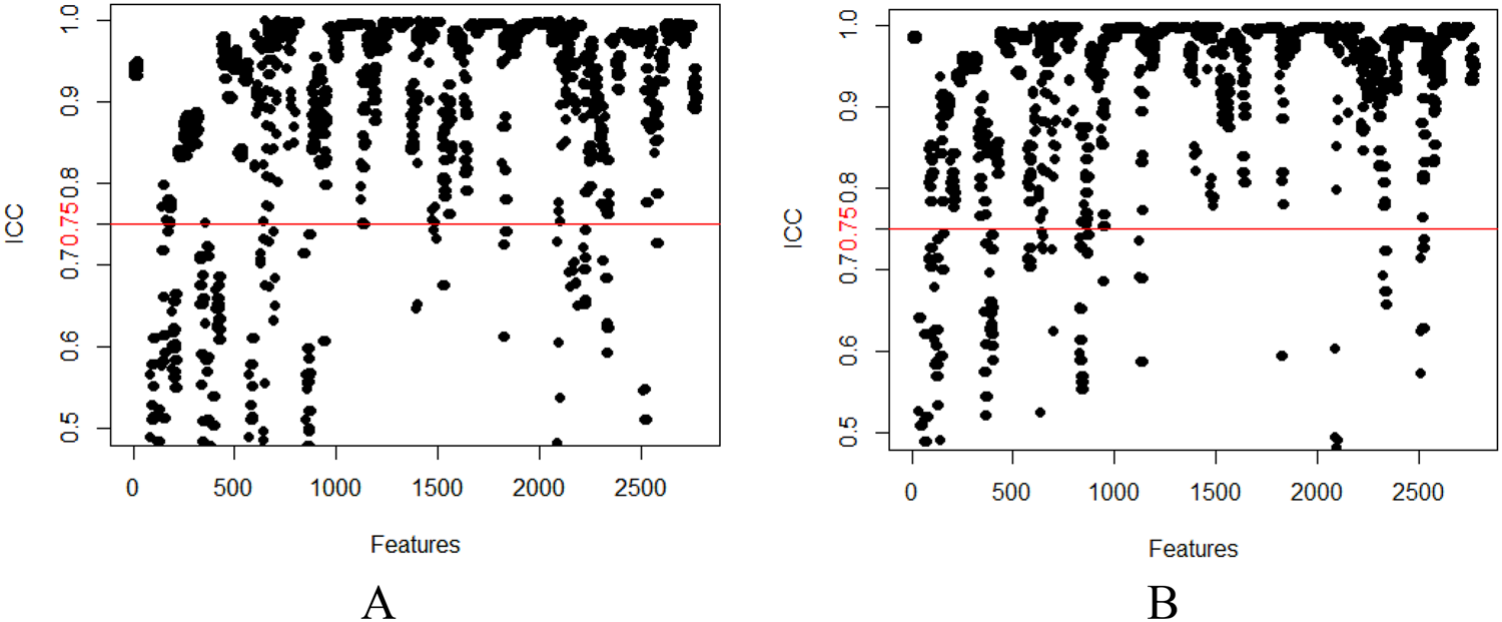
The consistency analyses in this study included intraobserver and interobserver consistency tests. Intragroup ICC/intergroup ICC values ≥ 0.75 indicated good agreement. **a** Intergroup ICC, **b** Intragroup ICC

**Fig. 4:**
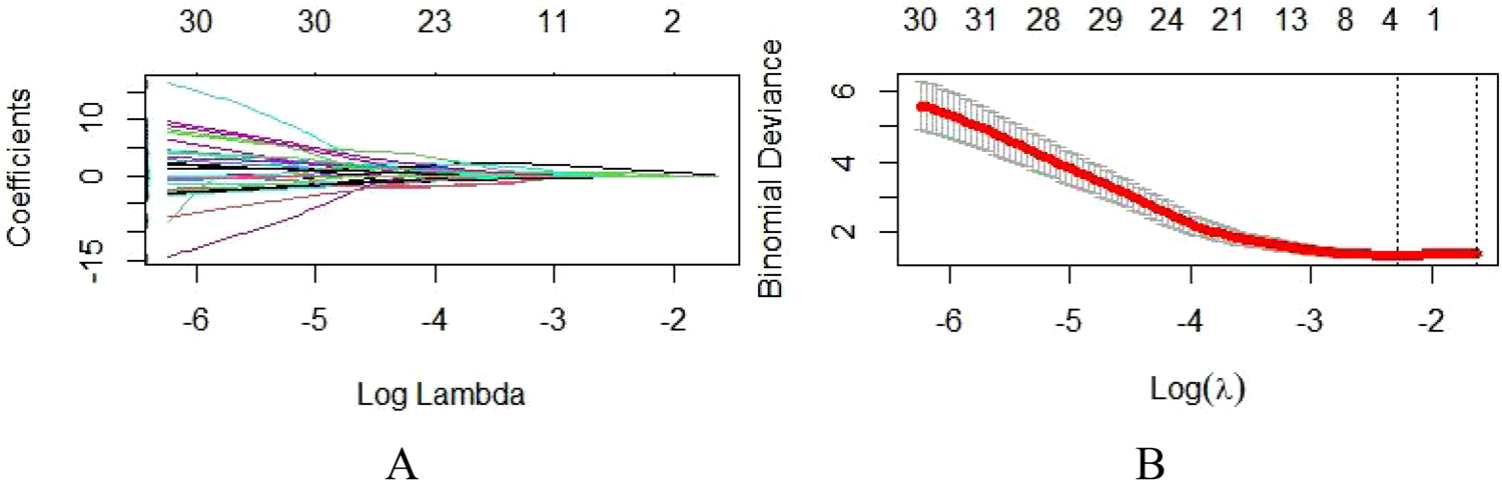
Radiomics feature selection using the LASSO regression analysis. **a** Coefficient convergence graph for optimal features, **b** Schematic diagram of LASSO

**Fig. 5:**
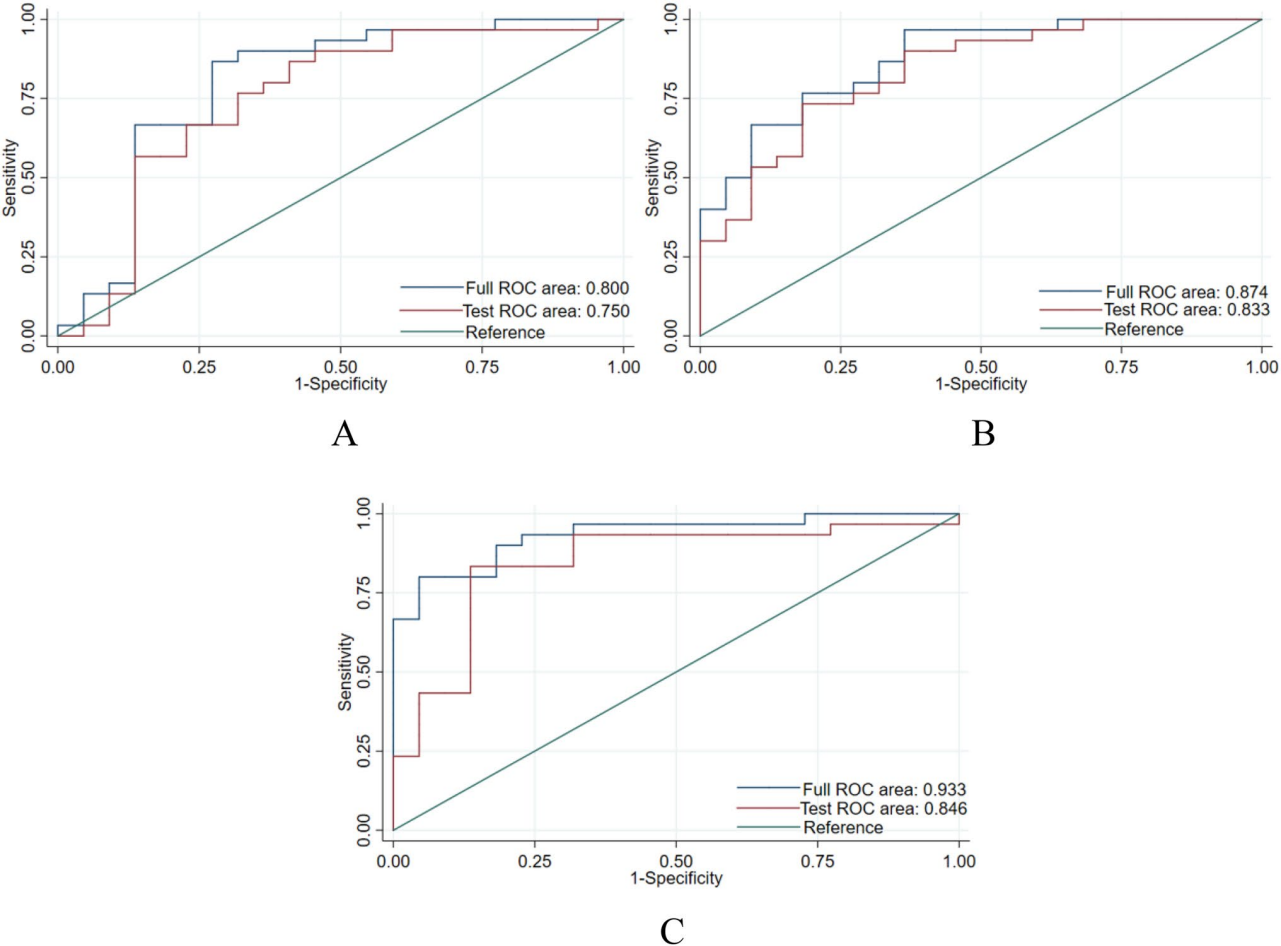
**a** ROC curves for the 10-fold cross-validation of the radiomics model, **b** ROC curves for the 10-fold cross-validation of the CR model, **c** ROC curves for the 10-fold cross-validation of the combined model

**Fig. 6:**
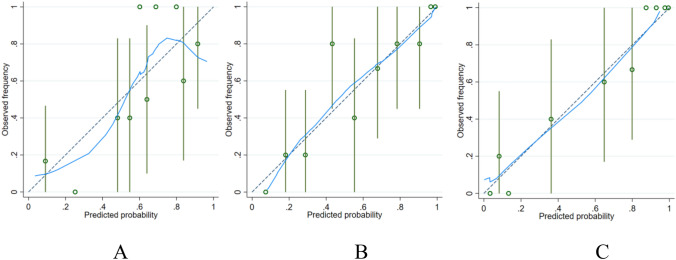
**a** Radiomics model calibration curve, **b** CR model calibration curve, **c** Combined model calibration curve; Calibration curve: predicted probability of the model and the actual probability; that is, the closer the nomogram is to the ideal curve, the better the fit of the model.

**Fig. 7:**
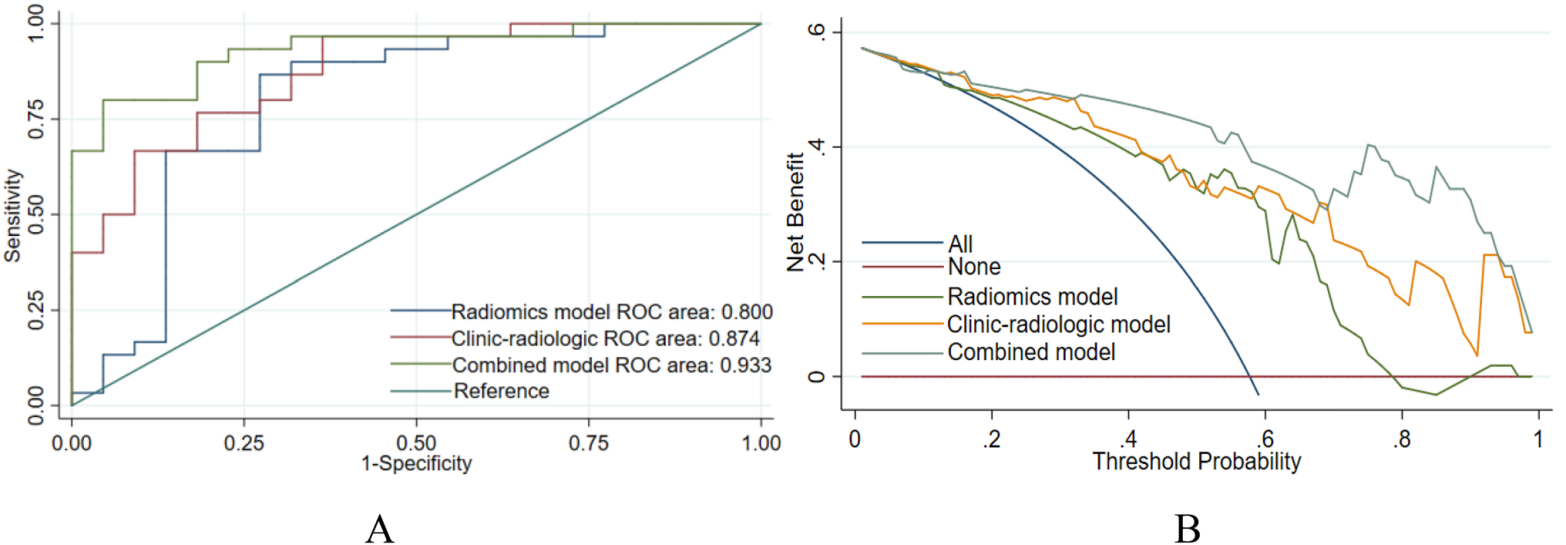
**a** ROC curves for various models, **b** Clinical DCA of 3 models; the *y*-axis represents the standardized net benefit, and the *x*-axis represents the high risk threshold; dark red (without Ang-2 expression) and blue (with Ang-2 expression) represent 2 extreme cases, and it is better if the curve is far from the 2 extreme cases.

The 52 patients in this study included 46 males and 6 females aged 29 to 70 years; the average age was 50.8 ± 10.9 years. Among the 52 HCC patients, high Ang-2 expression was found in 30 patients, and low Ang-2 expression was found in 22 patients. For each patient, 352 image features were extracted from the early arterial phase, late arterial phase, and portal venous phase, and 4 features (Table 1) were screened out after the intergroup consistency test (Fig. 3), univariate analysis, and LASSO dimensionality reduction (Fig. 4). Univariate and multivariate analyses indicated that D* and *f* (Table 2) were independent radiologic predictors of Ang-2 expression. Logistic regression models were established using the screened features. Tenfold cross-validation was used for validation (Fig. 5). Calibration curves were used to evaluate the model fit (Fig. 6). The AUCs for the radiomics model, the CR model and the combined model for the identification of Ang-2 expression were 0.800 (95% confidence interval (CI) 0.662–0.938), 0.874 (95% CI 0.781–0.968), and 0.933 (95% CI 0.868–0.998), respectively. The results of the DeLong test indicated that there was no significant difference in the AUC between the radiomics model and the CR model (*p* > 0.05) and that the AUC of the combined model was significantly greater than those of the other 2 models (*p* < 0.05). The results of DCA showed that the combined model outperformed the other 2 models and had the highest net benefit (Fig. 7).

**Table 1:** Selected radiomics features with descriptions

Feature type	Feature name	Coefficient
Arterial phase	GrayLevelCooccurenceMatrix25AutoCorrelation	0.35641360
	GrayLevelCooccurenceMatrix25ClusterShade	− 0.01175025
	GrayLevelCooccurenceMatrix25Entropy	0.96428990
Portal phase	GrayLevelCooccurenceMatrix25ClusterShade	− 0.10744972

**Table 2:** Univariate and multivariate analyses of clinic-radiologic characteristics for the evaluation of Ang-2 expression

Variables	Univariate analysis		Multivariate analysis	
	OR (95% CI)	*p* value	OR (95% CI)	*p* value
Sex (male/female)	0.704 (0.128–3.870)	0.686		
Age (years)	1.968 (0.568–6.824)	0.286		
INR (≤ 1.2/> 1.2)	0.789 (0.167–3.722)	0.765		
APTT (≤ 39/> 39 s)	0.648 (0.196–2.143)	0.477		
FIB (≤ 4/> 4 g/L)	4.200 (0.944–18.683)	0.059		
ATIII (≤ 130/> 130%)	1.700 (0.486–5.953)	0.407		
AST (≤ 40/> 40 U/L)	1.333 (0.399–4.457)	0.640		
ALT (≤ 50/> 50 U/L)	1.615 (0.509–5.123)	0.415		
ALP (≤ 125/> 125 U/L)	1.385 (0.443–4.327)	0.576		
GGT (≤ 60/> 60 U/L)	1.968 (0.568–6.824)	0.286		
ADA (≤ 25/> 25 U/L)	0.966 (0.261–3.573)	0.959		
AFU (≤ 40/> 40 U/L)	0.238 (0.026–2.201)	0.206		
5'NT (≤ 11/> 11 U/L)	2.211 (0.337–14.511)	0.409		
ChE (≤ 4000/> 4000 U/L)	0.652 (0.190–2.238)	0.497		
PA (≤ 150/> 150 mg/L)	0.933 (0.288–3.023)	0.908		
TP (≤ 65/> 65 g/L)	0.250 (0.073–0.850)	0.026		
ALB (≤ 40/> 40 g/L)	0.150 (0.029–0.763)	0.022		
GLOB (≤ 20/> 20 g/L)	1.333 (0.415–4.288)	0.629		
A/G (≤ 1.2/> 1.2)	0.297 (0.094–0.941)	0.039		
TBA (≤ 10/> 10 μmol/L)	1.667 (0.538–5.168)	0.376		
TBIL (≤ 26/> 26 μmol/L)	1.026 (0.205–5.132)	0.975		
DBIL (≤ 7/> 7 μmol/L)	0.632 (0.139–2.862)	0.551		
IBIL (≤ 17/> 17 μmol/L)	0.789 (0.167–3.722)	0.765		
AFP (≤ 400/> 400 µg/L)	3.701 (1.155–11.861)	0.028		
ADC	13.531 (0.940–194.679)	0.056		
D	10.927 (0.493–242.224)	0.130		
D^*^	1.062 (1.026–1.100)	0.001	1.053 (1.014–1.092)	0.007
f	1.169 (1.067–1.282)	0.001	1.160 (1.035–1.301)	0.011
PVTT	0.350 (0.096–1.278)	0.112		
Tumor size(≤ 5/> 5 cm)	1.371 (0.455–4.136)	0.575		
LNM	0.900 (0.212–3.828)	0.887		
Tumor boundary	0.429 (0.115–1.595)	0.206		

*INR* international normalized ratio, *APTT* activated partial thromboplastin time, *FIB* fibrinogen, *ATIII* antithrombin III, *AST* aspartate aminotransferase, *ALT* alanine aminotransferase, *ALP* alkaline phosphatase, *GGT* gamma glutamyl transpeptidase, *ADA* adenosine deaminase, *AFU* alpha-L-fucosidase, *5′NT* 5*′*-nucleotidase, *ChE* choline esterase, *PA* prealbumin, *TP* total protein, *ALB* albumin, *GLOB* globulin, *A/G* albumin-to-globulin ratio, *TBA* total bile acid, *TBIL* total bilirubin, *DBIL* direct bilirubin, *IBIL* indirect bilirubin, *AFP* alpha-fetoprotein, *PVTT* portal vein tumor thrombus, *LNM* lymph node metastasis.

## Discussion

The present study investigated the efficacy of predicting Ang-2 expression in HCC by preoperative DCE-MRI-based radiomics. In the whole cohort, high Ang-2 expression was found in 30 (57.7%) patients, which is consistent with the results (68.7%) of Chen et al. [14]. D^*^ and f were independent radiologic predictors of Ang-2 expression. The AUCs for the radiomics model, the CR model and the combined model for the identification of Ang-2 expression were 0.800, 0.874 and 0.933, respectively. The combined model outperformed the other 2 models and had the highest net benefit.

Angiogenesis is a key mechanism affecting the progression of neoplastic diseases. Ang-2 is an important protein capable of supporting angiogenesis under pathological conditions [17, 18]. Studies have shown [19] that Ang-2 may be a potential protein target for the nested metastasis of vessels encapsulated by tumor cluster (VETC)-positive HCC. Choi et al. [20] explored the potential of Ang-1, Ang-2, and VEGF levels in plasma as prognostic biomarkers from early to advanced stages of HCC. The results showed that Ang-2 levels had the highest predictive ability for overall survival (OS) in HCC patients, Ang-2 and alpha-fetoprotein (AFP) levels were independent factors for PFS, and Ang-2 was better than Ang-1 or VEGF as a prognostic biomarker for HCC, especially after local therapy. These findings suggest that detecting Ang-2 expression in HCC tissue can assist in providing personalized treatment for HCC patients.

The clinical application of IVIM-DWI allows the evaluation of HCC tumors at the microscopic level. Lee et al. [21] studied the relationship between IVIM-DWI parameters and microvessel density (MVD) in mouse colorectal cancer tissue, and the results showed that D^*^ and f values were significantly correlated with MVD. Ang-2 can promote angiogenesis in HCC tissue and is positively correlated with MVD [22]. Zheng et al. [23] investigated the correlations between IVIM-DWI parameters and Ang-2 in HCC and showed that D^*^ and f were significantly correlated with Ang-2 expression. In the present study, univariate and multivariate logistic regression analyses indicated that D^*^ and f were independent radiologic factors for predicting Ang-2 expression.

Radiogenomics is an advanced research topic in the fields of radiology and precision medicine. Radiogenomics is the study of the correlation of radiomic data with gene features and gene expression profiles [24]. Recent studies have shown that radiomics features can reflect biological processes that occur at the genetic and molecular levels [25]. Because there may be heterogeneity among HCC patients at the same disease stage, the choice of treatments for each HCC patient requires individualization [26]. HCC patients may benefit more from the prediction of treatment responses by radiomics. Combining traditional qualitative imaging and clinical data, quantitative imaging can be used to help identify many biomarkers to build predictive models to optimize the diagnosis, treatment selection, and treatment response monitoring of HCC [27]. Stefanie et al. [28] showed that radiomics features were correlated with the protein expression of the immunotherapy target programmed cell death-ligand 1 (PD-L1) (*r* = 0.41–0.47, *p* < 0.029) and the messenger RNA (mRNA) expression levels of programmed cell death-1 (PD-1) and cytotoxic T-lymphocyte-associated protein 4 (CTLA4) (*r* = − 0.48–0.47, *p* < 0.037), suggesting that MRI radiomics features can be used as noninvasive predictors of HCC immuno-oncology features, which can be helpful for the treatment stratification of HCC patients. Jia [29] et al. explored the value of a nomogram model that combined IVIM-DWI and radiomics features of rectal adenocarcinoma primary lesions in the preoperative assessment of nonenlarged lymph node metastasis (N-LNM), and the results showed that the nomogram model that combined IVIM-DWI parameters (D^*^ and *f*) and radiomics features had better evaluation performance (AUC = 0.864) than any other model in the training cohort. In addition, Zhang [30] showed that a nomogram based on clinical, IVIM-DWI and radiological parameters had high clinical value in predicting recurrence and disease-free survival (DFS) in patients with locally advanced cervical cancer (LACC) after concurrent chemoradiotherapy (CCRT), providing a reference for the prognostic assessment and individualized treatment of patients with cervical cancer. The present study used a radiomics model based on contrast-enhanced MRI sequences to noninvasively predict Ang-2 expression in HCC tissues, and a model established by combining radiomics features and CR factors obtained a higher predictive value and clinical net benefit.

The limitations of this study are as follows. First, although manual segmentation of ROIs was used in this study, inaccurate segmentation caused by unclear boundaries in some images was still possible. Second, this was a retrospective study, and therefore, selection bias may be present. Last, the sample size in this study was small, and this was a single-center study, thus lacking effective external validation.

In conclusion, DCE-MRI radiomics features can be used to build a model to predict Ang-2 expression in HCC patients, and the model that combined radiomics and CR characteristics had an improved prediction performance.

## Data Availability

The data generated and analyzed during this study are available from the corresponding author on reasonable request.

## Abbreviations

HCC: Hepatocellular carcinoma
Ang-2: Angiopoietin-2
DCE-MRI: Dynamic contrast‐enhanced magnetic resonance imaging
LASSO: Least absolute shrinkage and selection
IVIM-DWI: Intravoxel incoherent motion diffusion-weighted imaging
ICC: Intraclass correlation coefficient
OS: Overall survival
DFS: Disease-free survival
